# Evaluation of the anti-inflammatory activity of fisetin-loaded nanoparticles in an in vitro model of osteoarthritis

**DOI:** 10.1038/s41598-023-42844-1

**Published:** 2023-09-19

**Authors:** Zahra Nabizadeh, Mahmoud Nasrollahzadeh, Ali Akbar Shabani, Majid Mirmohammadkhani, Davood Nasrabadi

**Affiliations:** 1 Department of Medical Biotechnology, Faculty of Medicine, Semnan University of Medical Sciences, Semnan, Iran; 2 Department of Chemistry, Faculty of Science, University of Qom, P.O. Box 37185-359, Qom, Iran; 3 Department of Epidemiology and Biostatistics, Faculty of Medicine, Semnan University of Medical Sciences, Semnan, Iran

**Keywords:** Biotechnology, Rheumatology, Nanoscience and technology

## Abstract

Cartilage lesions, especially osteoarthritis (OA), are a common health problem, causing pain and disability in various age groups, principally in older adults and athletes. One of the main challenges to be considered in cartilage tissue repair is the regeneration of cartilage tissue in an active inflammatory environment. Fisetin has various biological effects including anti-inflammatory, antioxidant, apoptotic, and antiproliferative activities. The only disadvantages of fisetin in the pharmaceutical field are its instability and low solubility in aqueous media. This study is aimed at preparing chitosan (CS)-based nanoparticles to yield fisetin with improved bioavailability features. Then, the effect of fisetin-loaded nanoparticles (FNPs) on inflammatory responses in interleukin-1β (IL-1β) pretreated human chondrocytes has also been investigated. FNPs presented an average size of 363.1 ± 17.2 nm and a zeta potential of + 17.7 ± 0.1 mV with encapsulation efficiency (EE) and loading capacity (LC) of 78.79 ± 7.7% and 37.46 ± 6.6%, respectively. The viability of human chondrocytes was not affected by blank nanoparticles (BNPs) up to a concentration of 2000 μg/mL. In addition, the hemolysis results clearly showed that FNPs did not damage the red blood cells (RBCs) and had good hemocompatibility within the range investigated. FNPs, similar to fisetin, were able to inhibit the inflammatory responses induced by IL-1β such as the expression of interleukin-6 (IL-6) and tumor necrosis factor-α (TNF-α) while increasing the production of an anti-inflammatory cytokine such as interleukin-10 (IL-10). Overall, the in vitro evaluation results of the anti-inflammatory activity showed that FNPs can serve as delivery systems to transfer fisetin to treat inflammation in OA.

## Introduction

Osteoarthritis (OA), in which the entire joint is involved, is a degenerative disease, and inflammation makes a vital contribution to its onset and progression^1^. Due to the presence of inflammation at the damaged site, the regeneration of articular cartilage is clinically challenging^2^. The inflammatory cytokines with crucial shares in the development of OA include TNF-α, IL-1β, and IL-6^3^. The level of IL-1β, an important inflammatory mediator, increases in the synovial fluid and cartilaginous tissue of patients with OA^3^. IL-1β induces the expression of the degrading enzymes, such as matrix metalloproteinases (MMP-13) and a disintegrin and metalloproteinase with thrombospondin motifs (ADAMTS-5), and releases inflammatory mediators, such as cyclooxygenase-2 (COX-2) and inducible nitric oxide synthase (iNOS)^4^. The macrophages activated by TNF-α and IL-6 produce pro-inflammatory chemokines to maintain inflammation in many inflammatory diseases, such as OA^5^. Therefore, inhibiting these pro-inflammatory cytokines can be an effective curative target to treat OA. Fisetin (3,7,3′,4′-tetrahydroxyflavone), a plant flavonol found in several fruits and vegetables, such as strawberries, peaches, cucumbers, onions, mangoes, grapes, etc., has many biological activities including antioxidant, anti-inflammatory, anti-angiogenic, and anticancer^6^. One of the remarkable features of fisetin is the reduction of the IL-1ß-induced inflammatory effects through activating silent information regulator 1 (SirT1) in OA chondrocytes^3^. Therefore, fisetin can serve as a potential anti-inflammatory agent to treat OA^3^. However, the low solubility of fisetin in aqueous media and its limited half-life in the body limit the application of this valuable compound as a dietary supplement or medicinal compound^7^. In the treatment of OA, the curative effects mainly depend on the retention time of the drug in the joint^8^. Various studies have demonstrated that nanoparticles (NPs) increase the stability of a drug and control drug release in addition to reducing its side effects by delivering the drug to the target site^6^. In this study, CS was chosen for the synthesis of NPs due to its biocompatibility, biodegradability, non-antigenicity, non-toxicity, and polycationic properties^8^. Furthermore, the ionic gelation method using tripolyphosphate (TPP) anion was chosen to produce CS NPs because it is a simple and mild technique to prepare reproducible and monodisperse NPs^9^. Having synthesized FNPs, the particle size, zeta potential, polydispersity index (PDI), EE, LC, morphology, the release profile of fisetin from FNPs, cytocompatibility, and hemolysis assay were investigated. Finally, the inflammatory effects of FNPs on IL-1β-stimulated chondrocyte cells in vitro were investigated. This work is aimed at preparing CS NPs loaded with fisetin to improve its solubility to administer intra-articular, increase its retention time in the joint, improve its therapeutic effects, and minimize the corresponding side effects.

## Experimental

### Materials

TPP, CS (75–85% deacetylation degree, Mw 50–190 kDa), dimethyl sulfoxide (DMSO), acetic acid, Thiazolyl Blue Tetrazolium Bromide (MTT), and recombinant human IL-1β were obtained from Sigma-Aldrich Chemical Co. (St. Louis, MO, USA). Fisetin (purity > 98%) was obtained from NanoChemia Co. (Tehran, Iran). The human chondrocyte cell line (C28/I2) was purchased from the National Cell Bank of Iran (Pasteur Institute of Iran, Tehran, Iran). Penicillin/streptomycin, Fetal Bovine Serum (FBS), Dulbecco’s modified Eagle’s medium (DMEM)/Ham’s F12 medium, and 0.25% trypsin/EDTA were obtained from Gibco (UK).

### Synthesis of BNPs and FNPs

**Figure 1:**
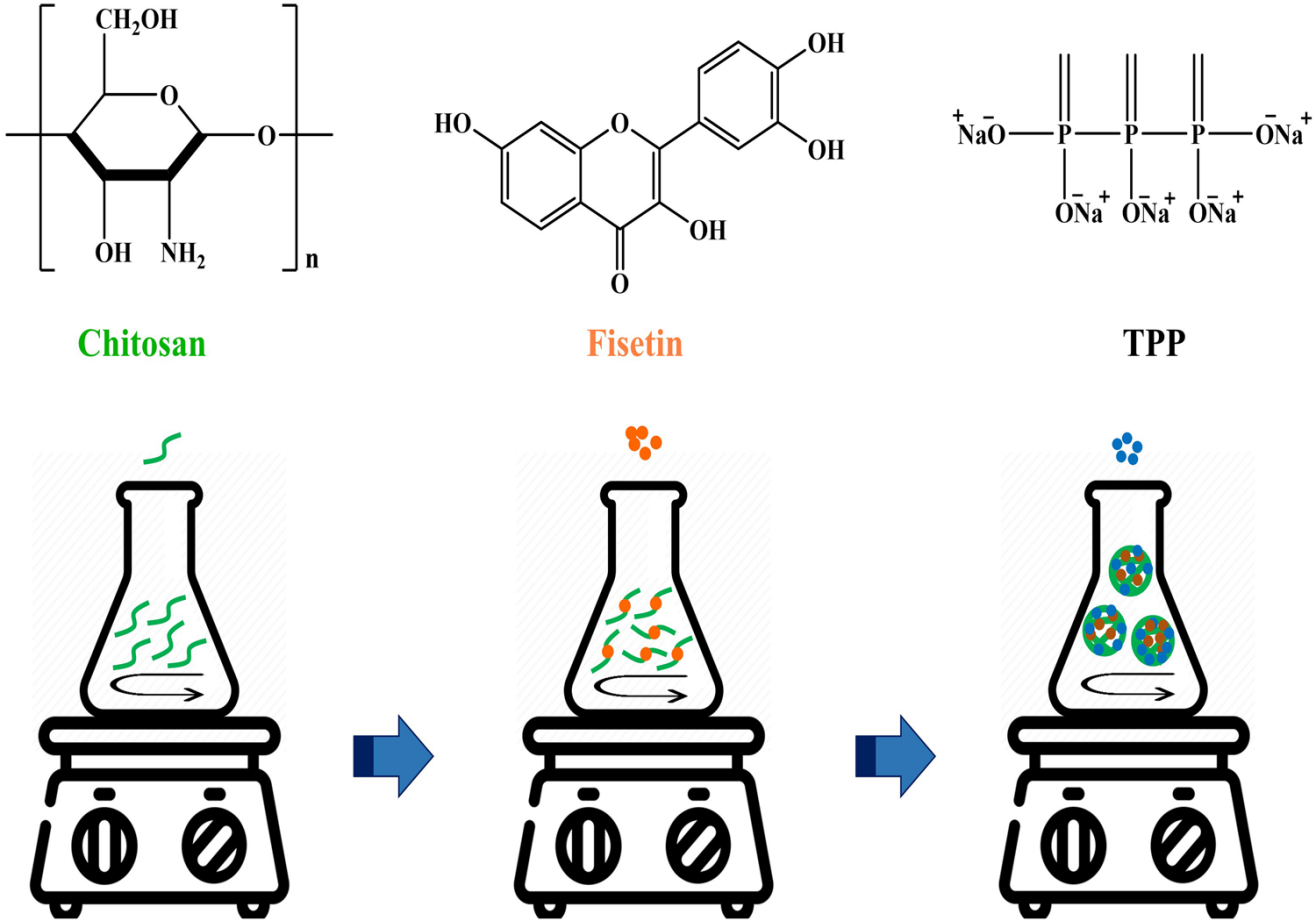
Chemical structures of CS, fisetin, and TPP and synthesis steps of FNPs.

BNPs and FNPs were synthesized based on the method reported by Zhang et al.^10^ with minor modifications (Fig. 1). A solution of CS (0.2%, w/v) was prepared in 1% acetic acid (v/v) under magnetic stirring (800 rpm) at ambient temperature overnight. The pH of the CS solution was adjusted to 5 using NaOH (2 M). Afterward, the CS solution was filtered through a 0.45 μm filter to separate any insoluble CS. TPP solution (4 mL) with a concentration of 1 mg/mL was added dropwise into the CS solution under stirring. The mixture obtained was then stirred for 20 min, followed by centrifugation at 12,000 rpm for 15 min (4 °C). The obtained NPs were freeze-dried and stored.

To form FNPs, the fisetin solution (1 mL) at a concentration of 3 mg/mL was prepared in a mixture of water and DMSO (2:1) and added dropwise to the CS solution (10 mL) under stirring (1 h). Next, the TPP solution (4 mL) was added dropwise to the CS solution containing fisetin. The elapsed time after adding the TPP solution was 15 min. To separate NPs, the resulting suspension was centrifuged at 12,000 rpm for 15 min (4 °C), washed twice with distilled water, and centrifuged again. The supernatant liquid was collected to determine LC and EE, while the precipitate was resuspended in distilled water and lyophilized to obtain FNPs.

### Determination of EE and LC

FNPs were isolated by centrifugation at 12,000 rpm, and the supernatant liquid was collected to determine LC and EE. The free fisetin in the supernatant was evaluated by spectrophotometry at 364 nm, which is the absorption maximum of fisetin. A standard curve was generated by a series of known concentrations of fisetin (Fig. S1). The amount of entrapped fisetin in FNPs was calculated by subtracting the amount of free fisetin (A_F_) in the supernatant from the total amount (A_T_) of fisetin used during the synthesis of FNPs. The percentages of EE and LC were found using Eqs. (1) and (2), respectively. In Eq. (2), A_M_ is the dry weight of FNPs.

1 $$ {\text{EE}}\left( \% \right) = \, \left[ {\left( {{\text{A}}_{{\text{T}}} - {\text{A}}_{{\text{F}}} } \right)/{\text{A}}_{{\text{T}}} } \right] \times {1}00 $$

2 $$ {\text{LC}}\left( \% \right) = \left[ {\left( {{\text{A}}_{{\text{T}}} - {\text{A}}_{{\text{F}}} } \right)/{\text{A}}_{{\text{M}}} } \right] \times {1}00 $$

### Characterization of NPs

Particle size, PDI, and zeta potential of NPs have been determined by Dynamic light scattering (DLS) (Malvern Instruments, UK) at 25 °C. The experiments were carried out in triplicate, and all the data were reported as the means ± standard deviation (SD). The morphology of NPs was investigated using atomic force microscopy (AFM) and scanning electron microscopy (SEM, TESCAN MIRA III, Czech Republic). For SEM, the suspensions of NPs were laid out on a glass plate and dried at room temperature. The samples were then coated with gold metal and examined. FTIR spectroscopy (model FTIR-8400s, Shimadzu Corp, Kyoto, Japan) was used to examine the formation of NPs. The freeze-dried NPs were blended with KBr and compressed into a pellet using a hydraulic press. All the spectra were recorded in the range of 400–4000 cm^−1^.

### Drug release

The release study of fisetin from FNPs was performed in PBS solution (pH 7.4 and pH 6) at 37 °C. A given amount of lyophilized FNPs containing 1 mg of fisetin was transferred to a clean tube with 2 mL of PBS buffer and shaken at 100 rpm at 37 °C. Next, 1 mL of the release media was collected at definite time intervals and replaced with the same volume of fresh PBS buffer (37 °C)^8^. Measurements were performed in triplicate to minimize the error variations. The drug concentration in the release media was found by quantifying the absorbance at 364 nm by UV–visible spectrophotometry and calculated using the standard calibration curve, as shown in Fig. S1.

### Biocompatibility study of NPs

MTT assay was used to investigate the potential cytotoxicity of BNP and FNPs on human chondrocytes. In short, the chondrocytes were cultured with a density of 6000 cells/well in 100 µL of DMEM/F12 media complemented with 10% FBS into 96-well plates. After 24 h, the media was completely withdrawn and substituted with 100 μL of fresh medium containing various concentrations of BNPs and FNPs. After incubation for 48 h, the medium was withdrawn, and PBS solution (pH 7.4) was used to wash the wells. Afterward, 100 μl of MTT solution (0.5 mg/mL, in PBS buffer) were added to each well and the cells were incubated for 3 h at 37 °C and 5% CO_2_. The MTT solution was substituted with 0.1 mL of DMSO to dissolve the formazan crystal. The absorbance at 570 nm was then recorded using a multi-mode microplate reader (BioTek Instruments, USA) and normalized to the value obtained for the control (non-treated cells). The cell toxicity was computed using Eq. (3).

3 $$ {\text{Toxicity}}\% \, = \left( {1 - \left( {\frac{mean\, OD\, of\, sample}{{mean\, OD\, of\, control}}} \right)} \right) \times 100 $$

### Hemolysis assay

A hemolytic assay was carried out based on the ISO 10993–4:2002 international standard method to evaluate the hemocompatibility of NPs. Fresh blood was obtained from a healthy human volunteer after institutional ethical approval and informed consent. The collected sample was centrifuged for 10 min at 3600 rpm. The RBCs were isolated from the plasma and rinsed with PBS (three times, pH 7.4). Certain amounts of lyophilized FNPs and BNPs were incubated with RBC suspension in a 96-well plate with three repetitions at 37 °C for 1 h. In addition, 1% Triton X-100 solution and 0.9% saline solution were considered as the positive (lysis ˷100%) and negative (lysis ˷0%) controls, respectively^6^. After incubation, the samples were centrifuged again at 3600 rpm (10 min) and the absorbance of the supernatant was quantified by photometric analysis at 414 nm, the maximum absorption of hemoglobin, using a microplate reader^11^. The hemolysis percentage was obtained using Eq. (4).

4 $$ {\text{Hemolysis}}\% = \left( {\frac{{mean\; OD\; of\; sample -  mean\; OD\; of\; negative\; control}}{{mean\; OD\; of\; positive\; control -  mean\; OD\, of\; negative\; control}}} \right) \times 100 $$

### Human chondrocyte treated with IL-1β

The human chondrocyte cell line (C28/I2) was seeded in DMEM/F12 medium complemented with 10% FBS and 1% penicillin/streptomycin solution. IL-1β is the most common cytokine used in OA modeling^12^. Therefore, the human chondrocytes were pretreated with IL-1β (10 ng/mL) for 5 h to mimic OA chondrocytes^13^. Afterward, chondrocytes were treated with FNPs for 48 h to investigate their effect on the expression of cartilage-related and inflammatory genes in OA chondrocytes.

### Gene expression analysis

The mRNA expression of inflammatory and cartilage-related genes was measured to survey the effect of FNPs on IL-1β pre-treated chondrocytes. RNA extraction from the treated human chondrocytes was conducted using the TRIzol reagent. RNA concentration was quantified using Thermo Scientific NanoDrop spectrophotometers. A cDNA synthesis kit (TaKaRa, Bio, Japan) was utilized to reverse-transcribe the total RNA to cDNA. Afterward, qRT-PCR was used to quantify the mRNA expression of SOX-9, COL2A1, SirT1, IL-10, TNF-α, and IL-6. The process of qPCR was comprised of the subsequent steps: 10 min at 94 °C followed by 35 cycles of 15 s at 95 °C, 20 s at 60 °C, and 30 s at 68 °C. The final reaction volume of 10 µL contained 0.5 µL of forward and reverse primers, 5 µL of SYBR Green Master Mix, and 4.5 µL of diluted cDNA. The level of target mRNA was computed by the 2^−ΔΔCT^ method, and GAPDH, a housekeeping gene, was utilized as an internal control. Table 1 includes all the specific sequences used in the reaction.

**Table 1:** Specific primers used in qRT-PCR reaction.

Gene	Forward primer	Reverse primer
SIRT1	5′-TAGACACGCTGGAACAGGTTGC-3′	5′-CTCCTCGTACAGCTTCACAGTC-3′
SOX-9	5′-GAGACTTCTGAACGAGAGCGA-3′	5′-CCGTTCTTCACCGACTTCCTC-3′
COL2A1	5′-GGAGCAGCAAGAGCAAGGAGAAG-3′	5′-TGGACAGCAGGCGTAGGAAGG-3′
TNF-α	5′-CTCTTCTGCCTGCTGCACTTTG-3′	5′-ATGGGCTACAGGCTTGTCACTC-3′
IL-10	5′-AGAATGCCTTTAATAAGCTCCA-3′	5′-GTCTATAGAGTCGCCACCC-3′
IL-6	5′-GAAAGCAGCAAAGAGGCACT-3′	5′-TTTCACCAGGCAAGTCTCCT-3′
GAPDH	5′-ACAACTTTGGTATCGTGGAAGG-3′	5′-GCCATCACGCCACAGTTTC-3′

### Statistical analysis

All the data obtained in this study were analyzed by one-way analysis of variance (ANOVA) in GraphPad Prism version 8.0.2.263. All the data were obtained in triplicate (n = 3) and expressed as mean ± SD. A *p*-value of less than 0.05 (*) was considered statistically significant. Meanwhile, all methods were performed according to the guidelines and regulations of the ethics committee of Semnan University of Medical Sciences.

### Ethics approval

The ethical committee of Semnan University of Medical Sciences, Faculty of Medicine, approved all experiments.

## Results and discussion

### Synthesis of NPs

Inflammation has a critical contribution to the progression of OA and is a limiting factor in its treatment^1^. Although fisetin has shown favorable anti-inflammatory activity, its low solubility in aqueous media limits its application in the pharmaceutical field. Various studies have demonstrated that NPs protect the drug from physiological degradation, improve drug bioavailability, and minimize the side effects on off-target tissues by delivering the drug to the target site^2^. CS is a biocompatible, biodegradable, and non-toxic compound used to synthesize NPs. In this study, FNPs were synthesized by a moderate technique, referred to as the ionic gelation method. CS was dissolved in 1% acetic acid (pH 5) under magnetic stirring to provide the -NH_3_^+^ site on the polymer. TPP dissolved in deionized water produces both OH^−^ and P_3_O_10_^5−^ ions, which are simultaneously present in TPP solution^14^. The ions (OH^−^ and P_3_O_10_^5−^) could compete to react ionically with -NH_3_^+^ present in CS by deprotonation or ionic crosslinking, respectively. Given their smaller size, OH^−^ ions easily penetrate CS. However, by acidifying the pH, only P_3_O_10_^5−^ ions exist in the solution^14^. Therefore, CS easily crosslinks with TPP. In this study, the pH of TPP solution (0.1%, w/v) was adjusted to 4 using acetic acid solution (1%, v/v). TPP was then added dropwise to the CS solution under stirring in a ratio of 2.5:1 (v/v, CS: TPP). For long-term storage and characterization of various parameters, NPs were freeze-dried and stored at 4 °C.

### Computation of EE and LC

Several factors affect EE, including the drug loading method, the concentration of CS and drug, the nature of the drug, the contact time of CS and drug, etc*.*^9^. There are two methods for drug loading: (a) drug incorporation during the preparation of NPs (incorporation method) and (b) drug absorption after the formation of NPs (absorption method)^6^. In this study, FNPs were prepared using the incorporation method. The fisetin solution was added dropwise to the CS solution under stirring before adding the TPP solution. After centrifugation, the supernatant of FNPs was used to calculate the amount of free fisetin using a spectrophotometer at 364 nm. The presence of phenolic hydroxyl groups on fisetin can affect EE by forming extensive hydrogen bonds with CS^9^. EE and LC, the key features to maintain the effective drug ratios in the intra-articular microenvironment, were 78.79 ± 7.7% and 37.46 ± 6.6%, respectively (Table 2). The EE percentage of FNPs in this study was parallel with the efficiency values described by Chuah et al. for curcumin encapsulated in CS nanoparticles (77.44 ± 0.2%)^15^.

**Table 2:** Nanoparticle size, PDI, zeta potential, EE, and LC of BNPs and FNPs.

	Size (nm)	PDI	Zeta potential (mV)	EE (%)	LC (%)
BNPs	214.3 ± 4.6	0.202 ± 0.001	35.7 ± 0.8	–	–
FNPs	363.1 ± 17.2	0.32 ± 0.01	17.7 ± 0.1	78.79 ± 7.7	37.46 ± 6.6

### Characterization of NPs

#### Size and morphology analysis of BNPs and FNPs

**Figure 2:**
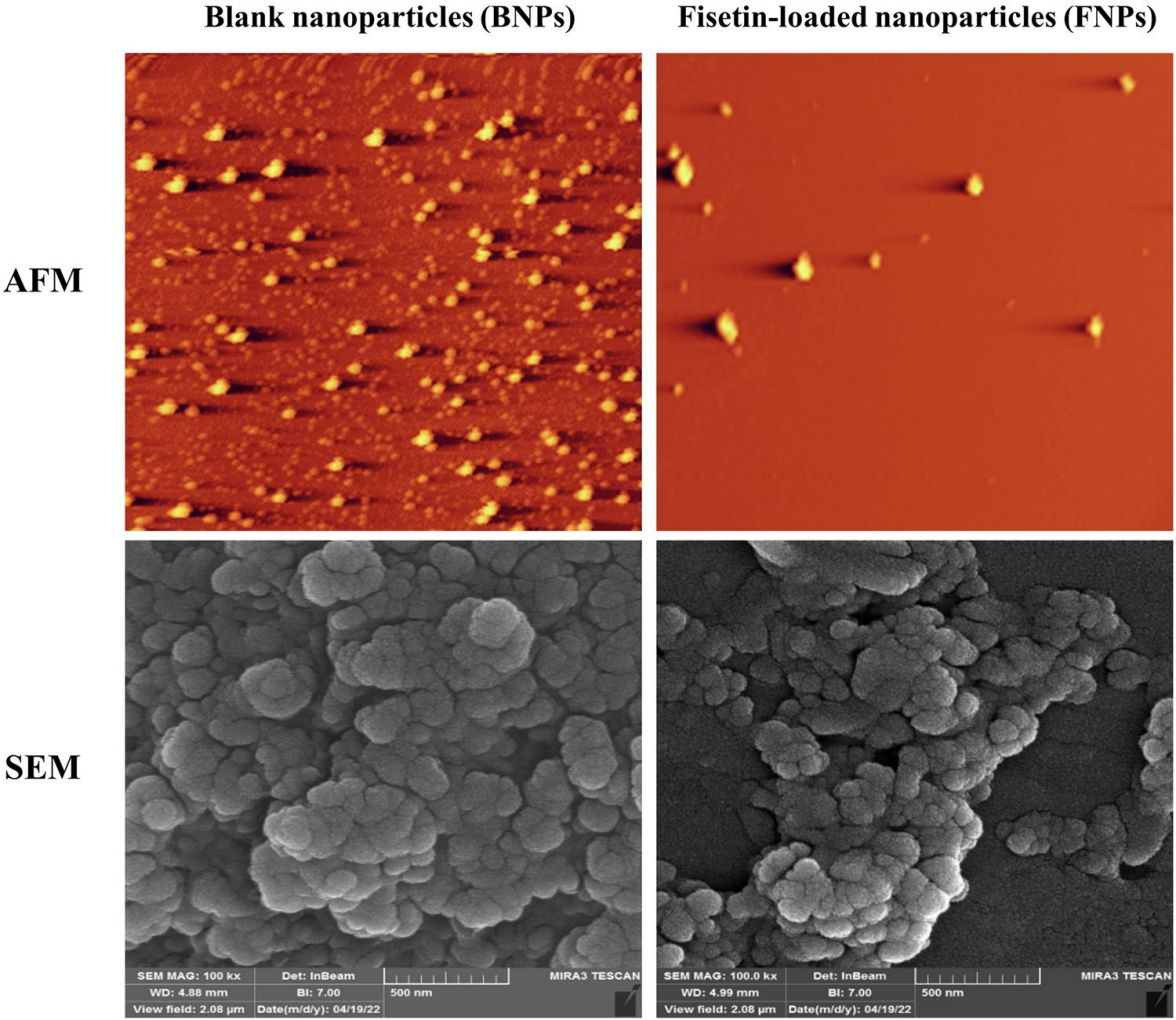
AFM and SEM images of BNPs and FNPs.

**Figure 3:**
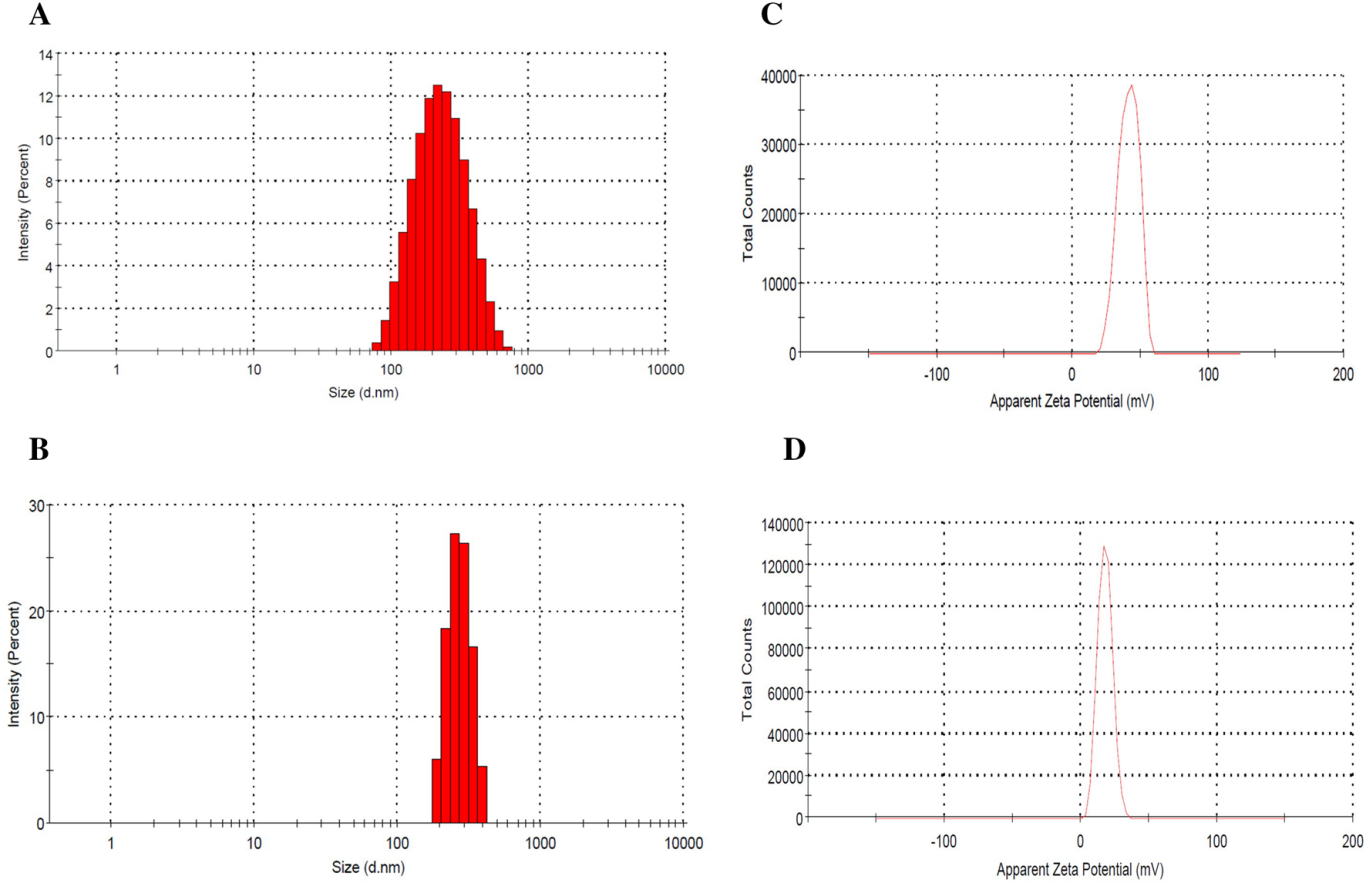
Characteristics of BNPs and FNPs: (**A**, **B**) size distribution of BNPs and FNPs, respectively; (**C**, **D**) zeta potential of BNPs and FNPs, respectively.

In NP systems, morphology, particle size, and size distribution are important factors affecting the stability of drug-loaded NPs, drug loading, and drug release^6^. The morphology of NPs was examined using AFM and SEM microscopes. The images clearly illustrate the sphericity of the NPs (Fig. 2), which is consistent with previous reports^8,16–18^. DLS was utilized to investigate the particle size, zeta potential, and PDI of NPs. Most studies^16,18–20^ using a ratio of 5:1 or 4:1 (w/w, CS: TPP) to prepare NPs have reported a positive zeta potential ranging from + 30 to + 39 mV, which is consistent with our results. BNPs had an average size and zeta potential of 214.3 ± 4.6 nm and + 35.7 ± 0.8 mV, respectively, while the corresponding values for FNPs were 363.1 ± 17.2 nm and + 17.7 ± 0.1 mV, respectively (Fig. 3 and Table 2). The loading of fisetin led to an increase in particle size and a decrease in zeta potential. The zeta potentials of both BNPs and FNPs were positive due to the cationic nature of CS (Table 2). However, the zeta potential of FNPs decreased compared with BNPs because of the neutralization of the amino groups of CS by interacting with the phenolic hydroxyl groups of fisetin^21^. NPs with a mean diameter in the 1–500 nm range are acceptable for injection^20,22^. Meanwhile, there is no limit on the average diameter of NPs used in intra-articular administration. Nowadays, NPs, microparticles, and hydrogels are used to improve the intra-articular delivery of specific drugs^2^. Hence, an average diameter of 363.1 ± 17.2 nm for FNPs is suitable for intra-articular injection. On the other hand, Kang et al.’s study showed that larger particles remained in the joint cavity longer^8^. Therefore, the increase in the retention time can very likely enhance the curative efficacy of a drug. A typical polydispersity of the suspension is between 0.1 and 0.5^23,24^. In our study, the PDI values of BNPs and FNPs were 0.202 ± 0.001 and 0.32 ± 0.01, respectively, which indicates that NPs are in monodisperse distribution mode^25^. The results of PDI were in agreement with those of previous studies^26–28^.

#### FTIR analysis

**Figure 4:**
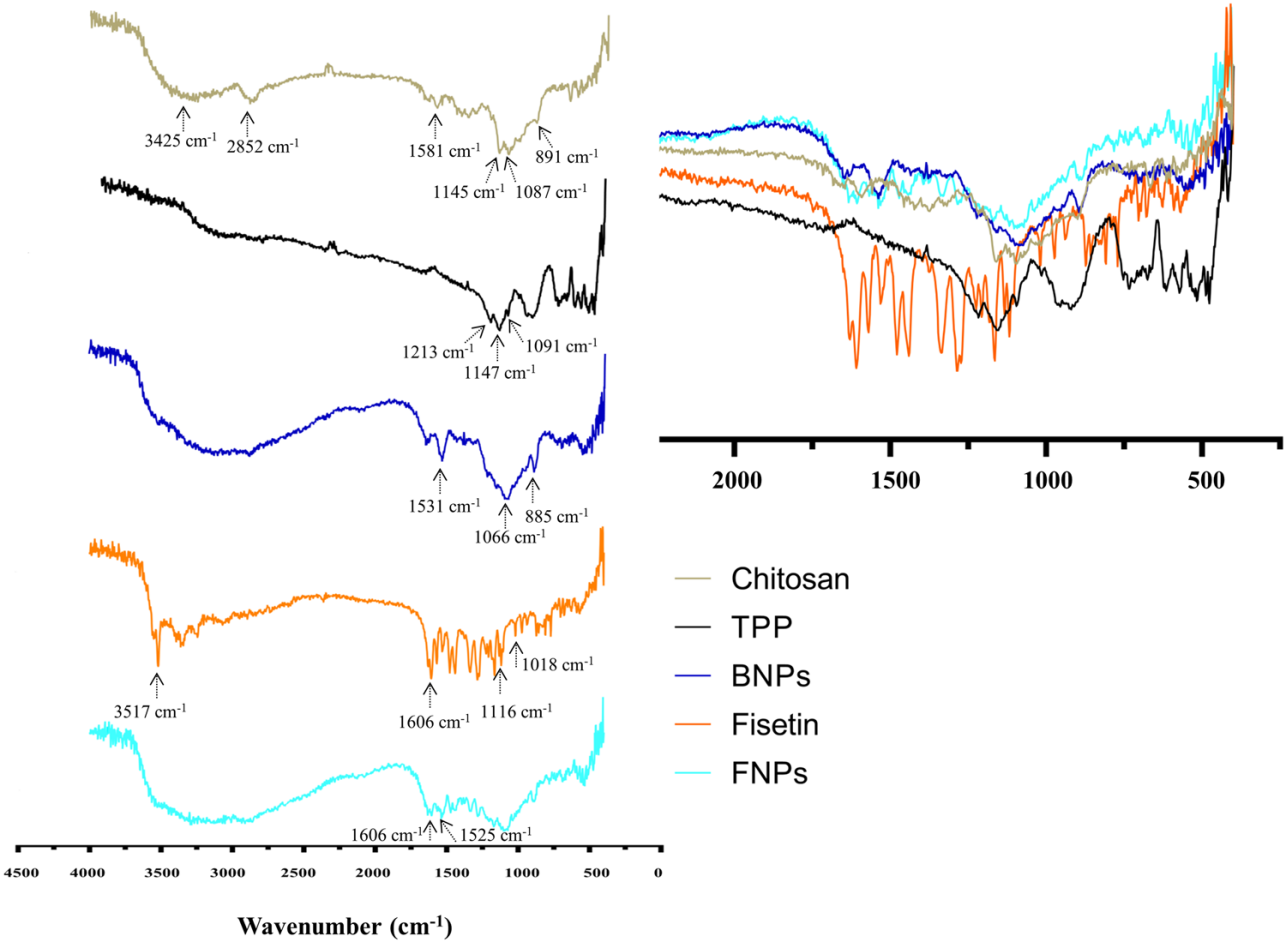
The FTIR spectra of CS, TPP, BNPs, fisetin, and FNPs: (**A**) full wavenumber range, (**B**) zoom of the range between 2250 and 250 cm^−1^.

FTIR spectroscopy was used to confirm the formation of NPs and examine the entrapment of fisetin in FNPs because this method can show the various peaks associated with the vibrations of chemical bonds and is sensitive to the conformational changes caused by breaking or forming intermolecular interactions. In the FTIR spectrum of CS (Fig. 4), the peak at 3425 cm^−1^ is ascribed to the stretching vibrations of –NH_2_ and –OH groups. The peak at 2852 cm^−1^ is related to the C–H stretching vibrations. In addition, the adsorption peak at 1581 cm^−1^ is due to the N–H bending of amine and amide II. Moreover, the peak at 1087 cm^−1^ corresponds to the C–O stretching vibration and the asymmetric stretch of C–O–C is confirmed by the peak at around 1145 cm^−129^. The FTIR spectrum of TPP (Fig. 4) shows the characteristic bands at about 1213 and 898 cm^−1^ which are associated with the stretching vibrations of the P=O and P–O–P bridge, respectively^30^. The peak at 1091 cm^−1^ is attributed to the symmetric and anti-symmetric stretching vibrations of PO_3_, and the band at 1147 cm^−1^ is related to the PO_2_ stretching^30^. Although the FTIR spectrum of BNPs is similar to that of pure CS, there are some differences. The peak at 3425 cm^−1^ is broadened because of the interaction of the TPP phosphate group with the NH_2_ group of pure CS and the increase of hydrogen bonding. The N–H bending from amide II at 1581 cm^−1^ has been enhanced and shifted to 1531 cm^−130^. For fisetin, the absorption peak at 3517 cm^−1^ corresponds to the –OH stretching mode, and the absorption bands at 1606 and 1525 cm^−1^ correspond to the C=C stretching vibrations^6^. Furthermore, the peaks at 1116 and 1018 cm^−1^ are related to the C–O–C group^6^. The differences between the FTIR spectra of BNPs and FNPs confirm that fisetin is encapsulated in FNPs. The bands at 1606 and 1525 cm^−1^, which correspond to the C=C stretching vibrations of the fisetin structure, are also present in FNPs.

#### Drug release

**Figure 5:**
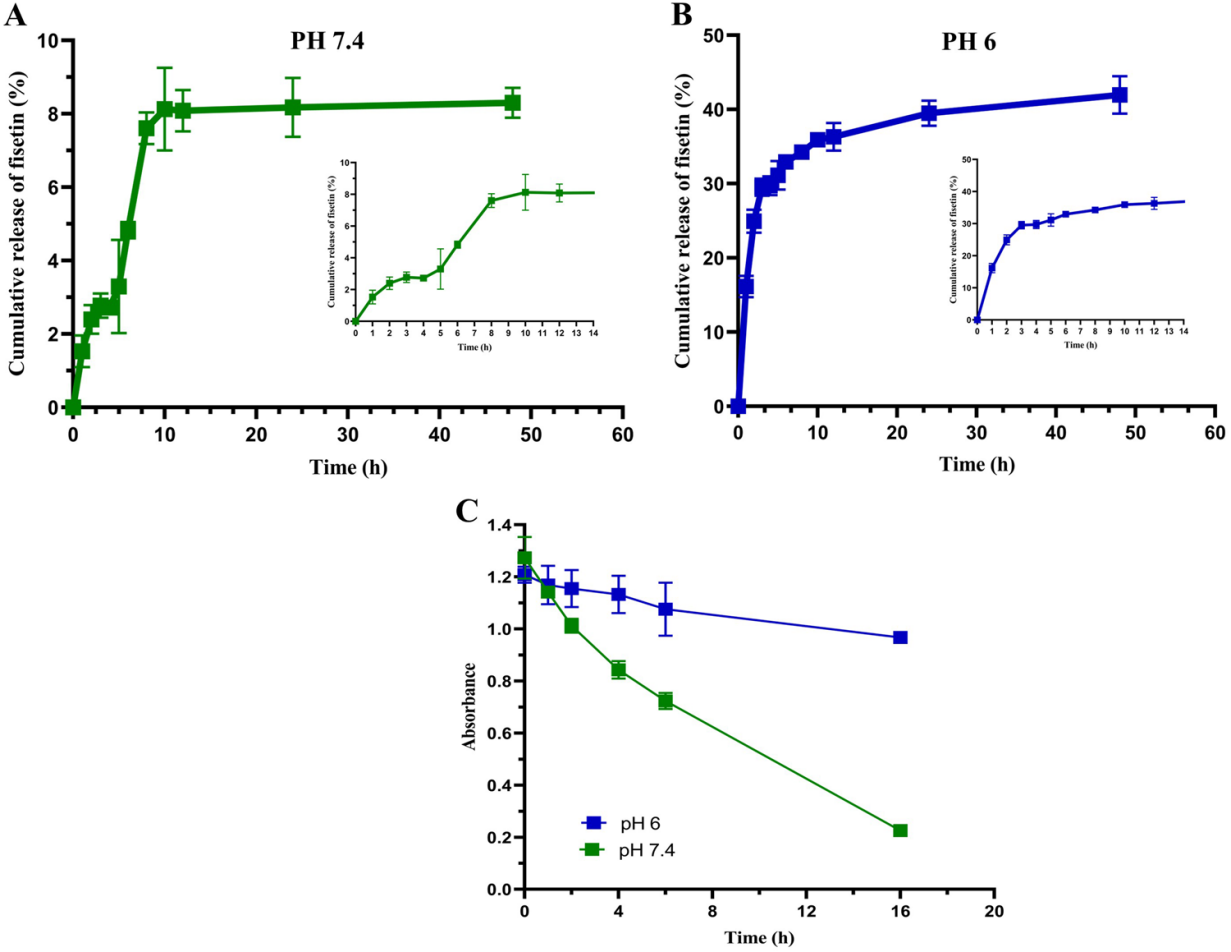
In vitro release study of fisetin from FNPs for 48 h in (**A**) pH 7.4 and (**B**) pH 6. (**C**) UV–vis absorbance of fisetin in PBS solutions with different pHs for different times at 37 °C.

To study the in vitro release of fisetin from NPs, the lyophilized FNPs were dispersed in PBS solution at different pHs (pH 7.4 and pH 6). pH 7.4, the optimal pH for the growth of most mammalian cells, was selected because the pH of the culture medium is about 7.4. pH 6 was chosen owing to the slightly acidic environment of the inflamed joint^31^. The study performed for 48 h represents a biphasic pattern (burst release and plateau) in both pHs (Fig. 5A,B). The initial burst release is most probably because of the adsorption of fisetin on the surface of NPs. The plateau step shows slow and sustained release assigned to the diffusion of fisetin entrapped within the polymer matrix. Of course, at this stage, the release due to erosion and degradation of NPs is also expected^9^. The release profile in PBS with pH 6 showed that the highest dissolution rate of 41% was detected within 48 h. However, the in vitro release data of fisetin from FNPs in PBS solution with pH 7.4 showed that only 8% of total fisetin was detected within 48 h. Since flavonoids contain sensitive chemical groups (hydroxyl) and structural elements (pyrone)^32^, a greater amount of fisetin has been presumably released and degraded in the PBS buffer. Therefore, a certain concentration of fisetin was prepared to investigate its degradation rate in PBS buffer (pH 7.4 and 6). After incubation time (1, 2, 4, 6, and16 h), the absorbance of fisetin at 364 nm was measured on a UV–Vis spectrophotometer (Shimadzu Co. Kyoto, Japan) using PBS buffer as the blank. The degradation study of fisetin in PBS with pH 7.4 showed that the absorbance of fisetin decreased in a time-dependent manner at 37 °C for 16 h (Fig. 5C). Nevertheless, the degradation rate was very slow at pH 6 compared to pH 7.4, as shown in Fig. 5C. The result was in agreement with the findings of Wang et al., who investigated the effects of pH, temperature, and the presence of protein in solutions on the degradation kinetics of fisetin in PBS buffer^32^. Their investigation demonstrated that the concentrations of fisetin at pH 7.4 decreased in a time-dependent manner. Indeed, increasing the pH from 6 to 7.4 enhanced the rate of fisetin degradation^32^. In addition, other studies reported that free flavonoids were unstable in aqueous solutions and sensitive to pH due to their chemical structure^33–37^. However, the presence of protein in the solution increases the stability^32,33^. In this regard, Wang et al. added some proteins into the PBS solution to investigate their effects on the degradation of fisetin^32^. Their results showed that the presence of proteins in the solution could effectively prevent fisetin degradation. Therefore, in our study, the higher toxicity of FNPs at low concentrations compared to BNPs indicates that more drug has been released into the environment than that shown by the release graph at pH 7.4 and the drug has been protected from degradation by the interaction with the proteins in the culture medium. Finally, many studies have demonstrated that drug incorporation inside the nanoparticle matrix can protect it from the external environment^6,8,38^.

#### In vitro cytotoxicity assay

**Figure 6:**
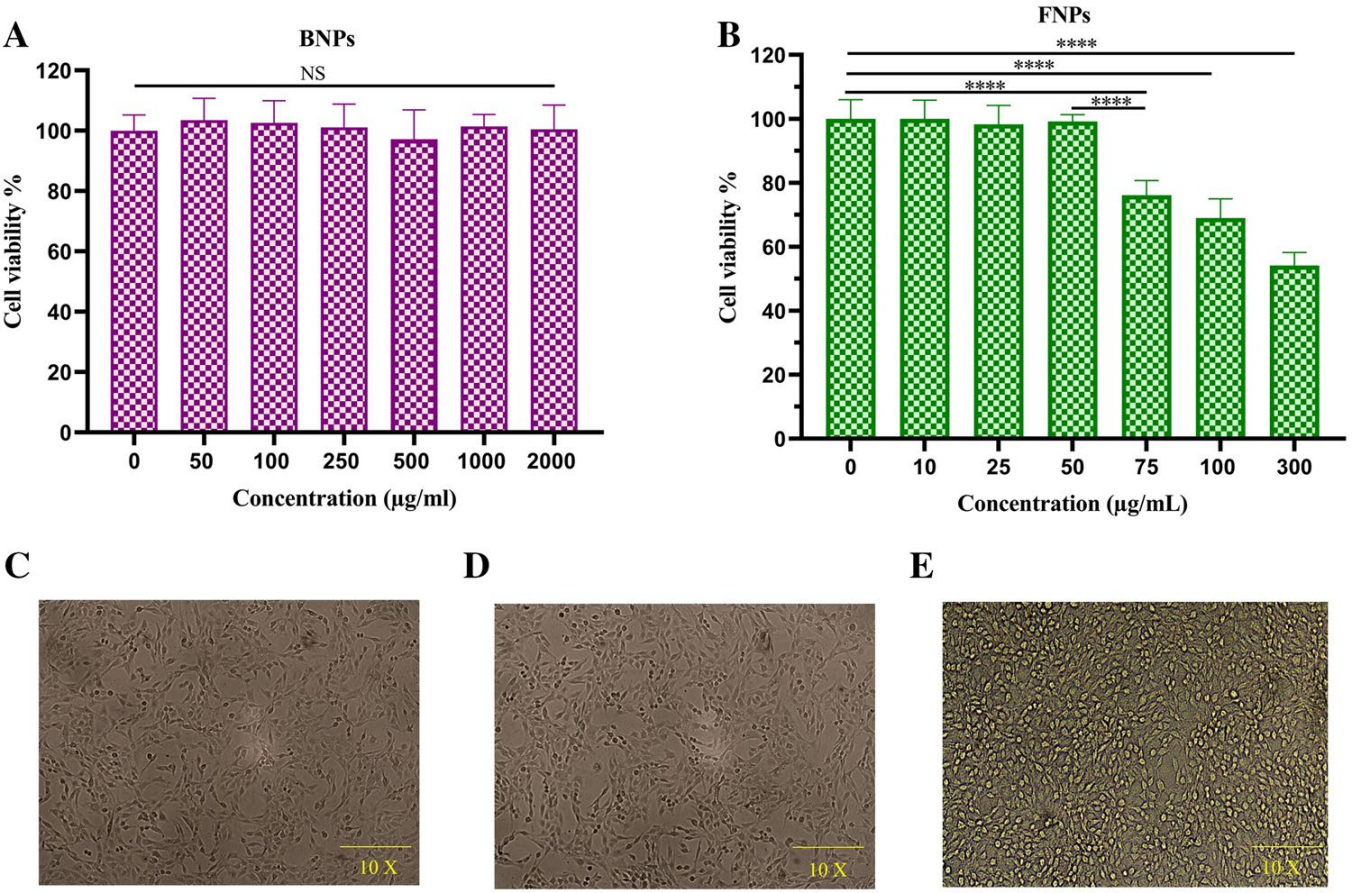
Cell viability of chondrocyte cells after 48 h incubation with (**A**) BNPs and (**B**) FNPs at various concentrations. Data were expressed as mean ± standard deviation (SD) (n = 4). Untreated chondrocyte cell morphology (**C**) and chondrocyte cell morphology after treatment with BNPs (2000 μg/mL) (**D**). The effect of cytotoxicity of FNPs on chondrocytes in monolayer culture (**E**). ****p < 0.0001. NS: not significant.

Cytotoxicity analysis of CS NPs as a drug delivery system is essential to confirm safety. Therefore, MTT assay was carried out to survey the cytotoxicity of BNPs on chondrocyte cells. Concentrations of BNPs ranging from 50 to 2000 μg/mL were designed to estimate their potential biocompatibility. The chondrocyte cell viabilities at concentrations of BNPs up to 2 mg/mL after 48 h of incubation were above 95%. The statistical comparison of the cell viability assay for BNPs did not show any significant differences between the groups and compared to the control group (0 concentration). This result was in line with those of previous reports^28,39^ and confirmed that this delivery system was safe for chondrocyte cells. The results of EE and LC % showed that a large amount of drug had been loaded into FNPs in this study. Although the percentage of EE and LC should be high to maintain an effective drug ratio in the intra-articular microenvironment and reduce the frequency of injections, it can cause toxicity in monolayer cultures. Therefore, the potential cytotoxicity of various concentrations of FNPs on chondrocytes was surveyed by MTT assay to find a concentration of FNPs, which was not toxic. The viability of chondrocytes was not affected by FNPs at the concentrations of up to 50 μg/mL, as shown in Fig. 6. As a result, the concentration of 0.05 mg/mL of FNPs was used in subsequent studies. Meanwhile, the study of the toxicity of free fisetin on chondrocytes by MTT assay showed its dose dependence (Fig. S2). The result was consistent with those reported in other studies^3,40–43^. Therefore, the difference in toxicity observed between BNPs and FNPs is due to the presence of fisetin in FNPs.

#### Hemolytic assay

**Figure 7:**
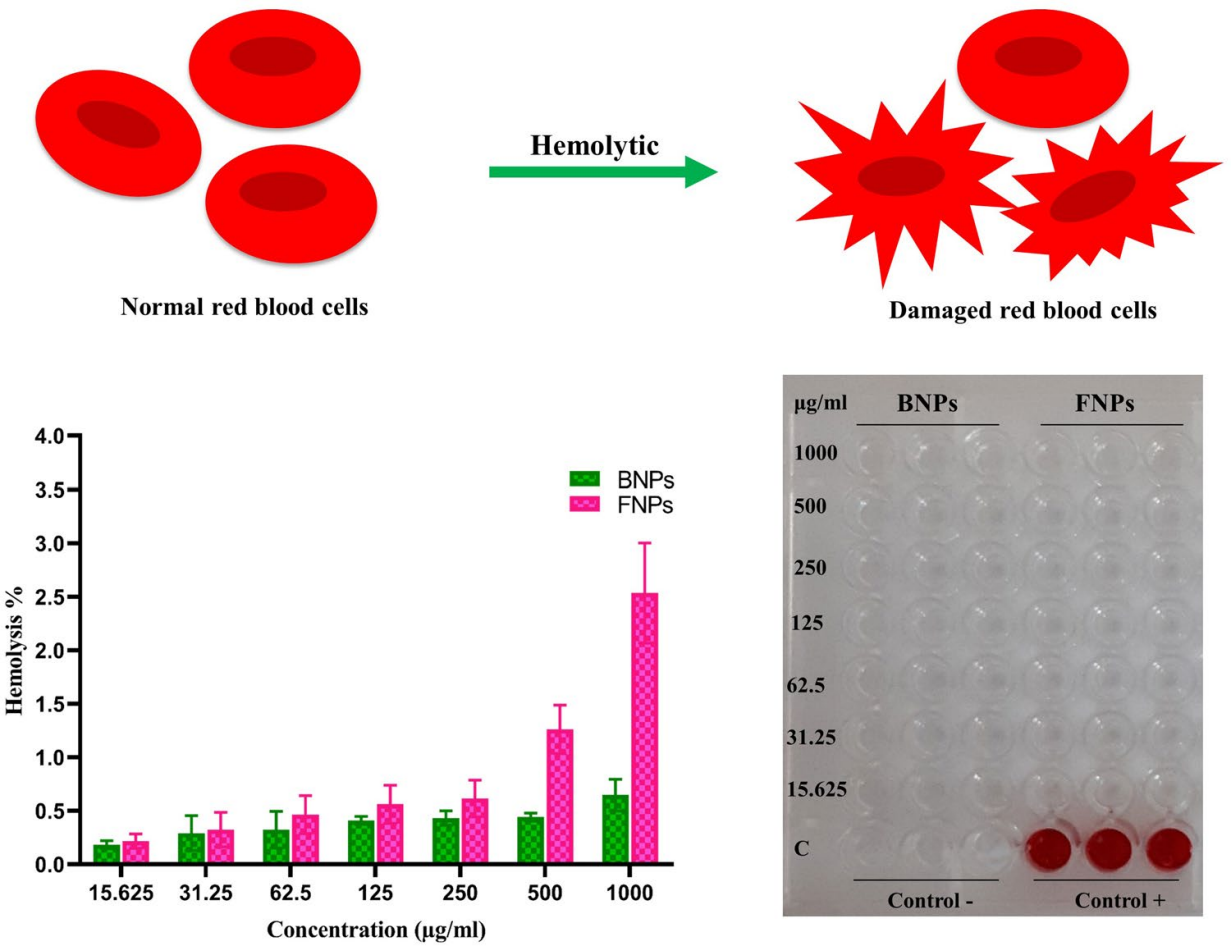
Evaluation of the hemolytic potential of various concentrations (15.625–1000 μg/mL) of BNPs and FNPs. 1% Triton X-100 solution (100% lysis) and 0.9% saline solution (0% lysis) were used as positive and negative controls, respectively.

NPs may change the membrane integrity of RBCs, leading to the leakage of hemoglobin (Hb) into the blood plasma. Nanoparticle characteristics such as size, surface charge, shape, and chemical composition can affect its hemolytic property^44^. Therefore, hemolytic assay was carried out to study the interaction of CS NPs with RBCs (Fig. 7). Hence, in this study, a fresh blood sample was treated with different concentrations (15.625–1000 μg/mL) of BNPs and FNPs. The results indicated that the hemolytic effects of BNPs and FNPs were lower than 0.65 and 2.5%, respectively, within the range of 15.625–1000 μg/mL. A hemolytic percentage above 5% is considered hemolytic^6^. Therefore, the hemolysis results clearly showed that both BNPs and FNPs did not damage RBCs and had good hemocompatibility within the range investigated. Our results were consistent with those reported by Dinesh et al., who investigated the hemocompatibility of CS/TPP NPs dispersed in different solutions (saline, acetic acid, and lactic acid)^45^. In general, the results of their investigation showed that CS/TPP NPs had acceptable blood compatibility upon dispersion in a biocompatible solvent such as saline and PBS buffer^9,45^.

#### Effect of FNPs on gene expression in chondrocytes pretreated with IL-1β

**Figure 8:**
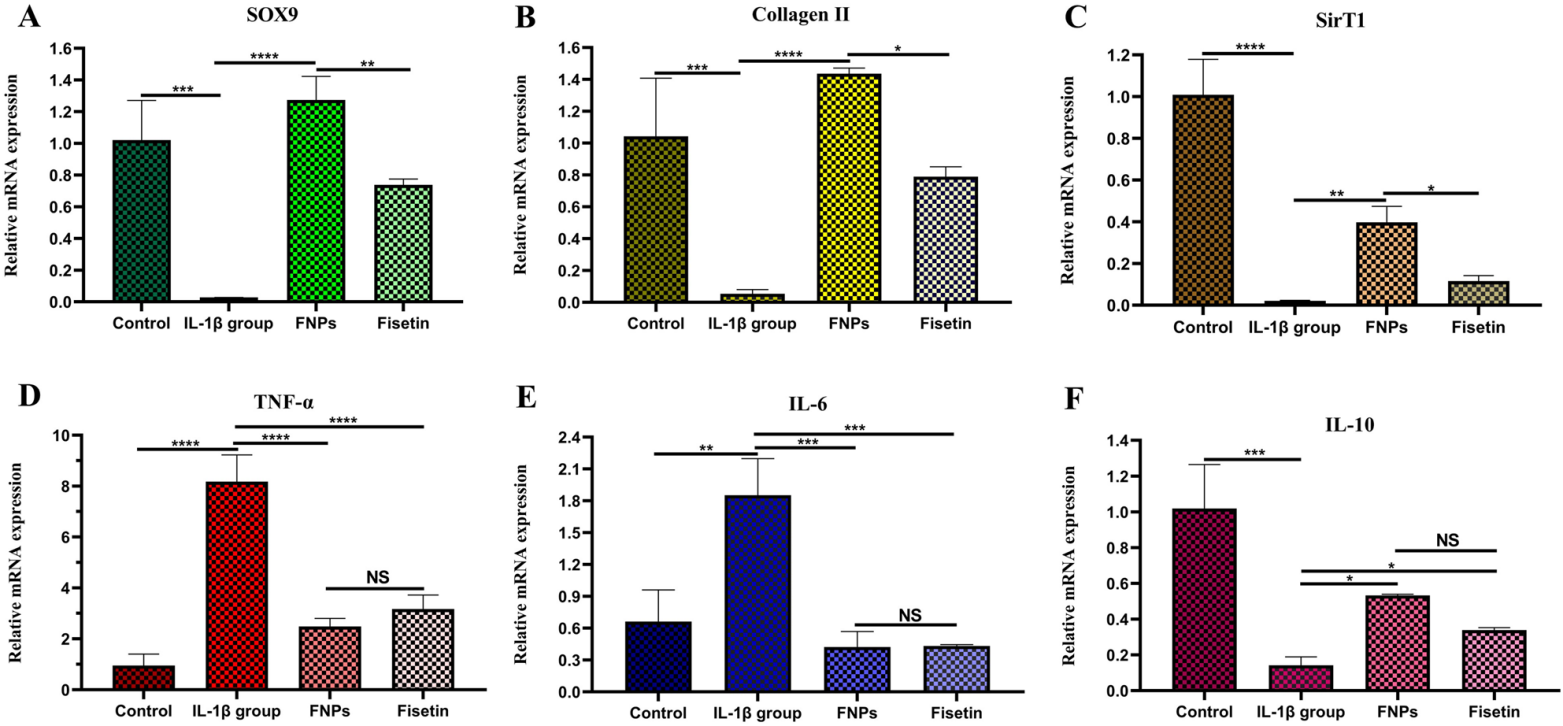
Effect of FNPs on the mRNA expression of inflammatory and cartilage-related genes in IL-1β pretreated human chondrocytes. Cells were stimulated for 5 h with IL-1β (10 ng/mL) and then treated with FNPs (50 μg/mL) or fisetin (7.5 μg/mL) for 48 h. The IL-1β group was stimulated with IL-1β (10 ng/mL) for 5 h only and the control group received no treatment. The mRNA expression levels of SOX9 (**A**), Collagen ΙΙ (**B**), SirT1 (**C**), TNF-α (**D**), IL-6 (**E**), and IL-10 (**F**) were assayed by qRT-PCR. The values are shown as mean ± SD (n = 3). *p < 0.05, **p < 0.01, ***p < 0.001, ****p < 0.0001. NS: not significant.

The effect of FNPs on the mRNA expression of inflammatory and cartilage-related genes in IL-1β pretreated human chondrocytes was surveyed. Cells were pretreated with IL-1β (10 ng/mL) for 5 h to mimic OA chondrocytes^13^, then treated with FNPs (50 μg/mL) or fisetin (7.5 μg/mL) for 48 h, followed by quantitative real-time PCR analysis. Many studies demonstrated that IL-1β remarkably down-regulated the mRNA expression of cartilage-related genes, such as Sox-9, COL2, and aggrecan^3,5^. Sox-9, a transcription factor, positively regulates the differentiation of chondrocytes by inducing the production of COL2 and aggrecan, the main components of cartilage extracellular matrix (ECM)^2^. Human chondrocyte cells pretreated with IL-1β showed a significant down-regulation in SOX-9 and COL2 mRNA expression compared to the untreated group (control group). However, FNPs significantly prevented the reduction of Sox-9 and COL2 mRNA expression in IL-1β stimulated chondrocyte cells (Fig. 8A,B). SirTI affects the differentiation and proliferation of chondrocytes and enhances their survival by up-regulating the cartilage-related genes such as COL2, Sox-9, and aggrecan and inhibiting apoptosis ^46,47^. Our results were in line with those of previous studies showing that the mRNA expression of SirT1 decreased in IL-1β pretreated human cells compared to the control group^3^. Nevertheless, the treatment with FNPs increased its levels in IL-1β stimulated human chondrocytes (Fig. 8C). TNF-α and IL-6, which activate macrophages to synthesize pro-inflammatory chemokines, make a critical contribution to maintaining the inflammation in the development of OA^3^. As shown in Fig. 8D,E, the stimulation of human chondrocytes with IL-1β for 5 h resulted in the up-regulation of TNF-α and IL-6 mRNA expression compared to the control group (non-treated cells). However, similar to fisetin, FNPs reduced TNF-α and IL-6 mRNA expression in IL-1β stimulated cells. Next, the effect of FNPs on the mRNA expression of IL-10, a well-known anti-inflammatory cytokine^48,49^, was investigated in IL-1β stimulated human chondrocytes (Fig. 8F). The results indicated that although the IL-10 level was decreased in chondrocytes stimulated with IL-1β compared with the untreated group (control group), the treatment with FNPs, like fisetin^48,49^, increased its level in IL-1β pretreated human chondrocytes. In general, these results showed that FNPs can inhibit the inflammatory responses induced by IL-1β, including the expression of TNF-α, and IL-6, and reduce the degradation of Sox-9, COL2, and SirT1 in OA chondrocyte cells.

## Conclusions

In this study, FNPs with homogeneous particle size, distribution, and suitable drug LC were successfully prepared for fisetin delivery. Since there are no limits on the average diameter of NPs used in the intra-articular injection, an average diameter of 363.1 ± 17.2 nm for FNPs can be appropriate for the intra-articular injection. The results showed that FNPs, like fisetin, can increase the secretion of IL-10, an anti-inflammatory cytokine, while repressing the synthesis of pro-inflammatory cytokines such as TNF-α and IL-6. To our knowledge, this is the first report regarding the loading of fisetin into NPs to treat the inflammation in OA. The intra-articular injection of new drug formulations can maintain the drug in synovial fluid at clinically relevant concentrations and reduce its uptake into the systemic circulation. Therefore, further evaluations with an animal model of OA are needed to prove whether FNPs can improve drug bioavailability and increase its retention time in the joint cavity. Since fisetin has several biological properties including anticancer, antioxidant, anti-angiogenic, and anti-inflammatory activities^6^, FNPs can also be used to investigate their therapeutic effects on the in vitro and in vivo models of other diseases such as cancer.

### Supplementary Information

Supplementary Information.

## Data Availability

The datasets used and/or analyzed during the current study are available from the corresponding author upon reasonable request.

## References

[1] Koh RH, Jin Y, Kim J, Hwang NS (2020). Inflammation-modulating hydrogels for osteoarthritis cartilage tissue engineering. Cells.

[2] Nabizadeh Z (2022). Micro-and nanotechnology in biomedical engineering for cartilage tissue regeneration in osteoarthritis. Beilstein J. Nanotechnol..

[3] Zheng W (2017). Fisetin inhibits IL-1β-induced inflammatory response in human osteoarthritis chondrocytes through activating SIRT1 and attenuates the progression of osteoarthritis in mice. Int. Immunopharmacol..

[4] Chabane N (2008). Histone deacetylase inhibitors suppress interleukin-1β-induced nitric oxide and prostaglandin E2 production in human chondrocytes. Osteoarth. Cartil..

[5] Feldmann M, Brennan FM, Maini RN (1996). Role of cytokines in rheumatoid arthritis. Annu. Rev. Immunol..

[6] Ghosh P (2016). Preparation of albumin based nanoparticles for delivery of fisetin and evaluation of its cytotoxic activity. Int. J. Biol. Macromol..

[7] Pawar A, Singh S, Rajalakshmi S, Shaikh K, Bothiraja C (2018). Development of fisetin-loaded folate functionalized pluronic micelles for breast cancer targeting. Artif. Cells Nanomed. Biotechnol..

[8] Kang ML, Ko J-Y, Kim JE, Im G-I (2014). Intra-articular delivery of kartogenin-conjugated chitosan nano/microparticles for cartilage regeneration. Biomaterials.

[9] Bugnicourt L, Ladavière C (2016). Interests of chitosan nanoparticles ionically cross-linked with tripolyphosphate for biomedical applications. Prog. Polym. Sci..

[10] Zhang Y, Yang Y, Tang K, Hu X, Zou G (2008). Physicochemical characterization and antioxidant activity of quercetin-loaded chitosan nanoparticles. J. Appl. Polym. Sci..

[11] Eivazzadeh-Keihan R (2022). A novel, bioactive and antibacterial scaffold based on functionalized graphene oxide with lignin, silk fibroin and ZnO nanoparticles. Sci. Rep..

[12] Johnson CI, Argyle DJ, Clements DN (2016). In vitro models for the study of osteoarthritis. Vet. J..

[13] Wang S-J, Qin J-Z, Zhang T-E, Xia C (2019). Intra-articular injection of kartogenin-incorporated thermogel enhancing osteoarthritis treatment. Front. Chem..

[14] Mi FL, Shyu SS, Lee ST, Wong TB (1999). Kinetic study of chitosan-tripolyphosphate complex reaction and acid-resistive properties of the chitosan-tripolyphosphate gel beads prepared by in-liquid curing method. J. Polym. Sci. Part B: Polym. Phys..

[15] Chuah LH, Roberts CJ, Billa N, Abdullah S, Rosli R (2014). Cellular uptake and anticancer effects of mucoadhesive curcumin-containing chitosan nanoparticles. Colloids Surf. B.

[16] Pan C (2020). Study on the relationship between crosslinking degree and properties of TPP crosslinked chitosan nanoparticles. Carbohyd. Polym..

[17] Sang Z (2020). Comparison of three water-soluble polyphosphate tripolyphosphate, phytic acid, and sodium hexametaphosphate as crosslinking agents in chitosan nanoparticle formulation. Carbohyd. Polym..

[18] Seyedebrahimi R, Razavi S, Varshosaz J (2020). Controlled delivery of brain derived neurotrophic factor and gold-nanoparticles from chitosan/TPP nanoparticles for tissue engineering applications. J. Cluster Sci..

[19] Mariano KCF (2019). Influence of chitosan-tripolyphosphate nanoparticles on thermosensitive polymeric hydrogels: Structural organization, drug release mechanisms and cytotoxicity. Int. J. Polym. Mater. Polym. Biomater..

[20] Mahmood MA, Madni A, Rehman M, Rahim MA, Jabar A (2019). Ionically cross-linked chitosan nanoparticles for sustained delivery of docetaxel: fabrication, post-formulation and acute oral toxicity evaluation. Int. J. Nanomed..

[21] Ilk S, Sağlam N, Özgen M, Korkusuz F (2017). Chitosan nanoparticles enhances the anti-quorum sensing activity of kaempferol. Int. J. Biol. Macromol..

[22] Sun M (2020). Rebamipide-loaded chitosan nanoparticles accelerate prostatic wound healing by inhibiting M1 macrophage-mediated inflammation via the NF-κB signaling pathway. Biomater. Sci..

[23] Kong D, Kusrini E, Wilson LD (2021). Binary pectin-chitosan composites for the uptake of lanthanum and yttrium species in aqueous media. Micromachines.

[24] Pedroso-Santana S, Fleitas-Salazar N (2020). Ionotropic gelation method in the synthesis of nanoparticles/microparticles for biomedical purposes. Polym. Int..

[25] Wu J, Wang Y, Yang H, Liu X, Lu Z (2017). Preparation and biological activity studies of resveratrol loaded ionically cross-linked chitosan-TPP nanoparticles. Carbohyd. Polym..

[26] Weng J, Tong HH, Chow SF (2020). In vitro release study of the polymeric drug nanoparticles: development and validation of a novel method. Pharmaceutics.

[27] Safari F, Mirzaeei S, Mohammadi G (2021). Development of chitosan-tripolyphosphate nanoparticles as glycopeptide antibiotic reservoirs and ex vivo evaluation for their potential to enhance the corneal permeation in ocular drug delivery. Pharm. Sci..

[28] Jain A, Thakur K, Sharma G, Kush P, Jain UK (2016). Fabrication, characterization and cytotoxicity studies of ionically cross-linked docetaxel loaded chitosan nanoparticles. Carbohyd. Polym..

[29] Antoniou J (2015). Physicochemical and morphological properties of size-controlled chitosan–tripolyphosphate nanoparticles. Colloids Surf. A.

[30] de Carvalho FG (2019). Synthesis and characterization of TPP/chitosan nanoparticles: Colloidal mechanism of reaction and antifungal effect on C. albicans biofilm formation. Mater. Sci. Eng. C.

[31] Xiong F (2020). pH-responsive and hyaluronic acid-functionalized metal–organic frameworks for therapy of osteoarthritis. J. Nanobiotechnol..

[32] Wang J, Zhao X-H (2016). Degradation kinetics of fisetin and quercetin in solutions as effected by pH, temperature and coexisted proteins. J. Serb. Chem. Soc..

[33] Xiao J, Högger P (2015). Stability of dietary polyphenols under the cell culture conditions: Avoiding erroneous conclusions. J. Agric. Food Chem..

[34] Zenkevich IG (2007). Identification of the products of oxidation of quercetin by air oxygen at ambient temperature. Molecules.

[35] Makris DP, Rossiter JT (2000). Heat-induced, metal-catalyzed oxidative degradation of quercetin and rutin (quercetin 3-O-rhamnosylglucoside) in aqueous model systems. J. Agric. Food Chem..

[36] Barnes JS, Schug KA (2014). Oxidative degradation of quercetin with hydrogen peroxide using continuous-flow kinetic electrospray–ion trap–time-of-flight mass spectrometry. J. Agric. Food Chem..

[37] Friedman M, Jürgens HS (2000). Effect of pH on the stability of plant phenolic compounds. J. Agric. Food Chem..

[38] Kumar GV, Su C-H, Velusamy P (2016). Preparation and characterization of kanamycin-chitosan nanoparticles to improve the efficacy of antibacterial activity against nosocomial pathogens. J. Taiwan Inst. Chem. Eng..

[39] Iswanti FC (2019). Preparation, characterization, and evaluation of chitosan-based nanoparticles as CpG ODN carriers. Biotechnol. Biotechnol. Equip..

[40] Kumar RM (2023). Fisetin in cancer: Attributes, developmental aspects, and nanotherapeutics. Pharmaceuticals.

[41] Yousefzadeh MJ (2018). Fisetin is a senotherapeutic that extends health and lifespan. EBioMedicine.

[42] Imran M (2021). Fisetin: An anticancer perspective. Food Sci. Nutr..

[43] Saas J, Lindauer K, Bau B, Takigawa M, Aigner T (2004). Molecular phenotyping of HCS-2/8 cells as an in vitro model of human chondrocytes. Osteoarth. Cartil..

[44] Sasidharan A (2012). Hemocompatibility and macrophage response of pristine and functionalized graphene. Small.

[45] Nadesh R, Narayanan D, Pr S, Vadakumpully S, Mony U, Koyakkutty M, Nair SV, Menon D (2013). Hematotoxicological analysis of surface-modified and -unmodified chitosan nanoparticles. J. Biomed. Mater. Res. A.

[46] Takayama K (2009). SIRT1 regulation of apoptosis of human chondrocytes. Arthritis Rheum..

[47] Dvir-Ginzberg M, Mobasheri A, Kumar A (2016). The role of sirtuins in cartilage homeostasis and osteoarthritis. Curr. Rheumatol. Rep..

[48] Song B (2022). Effects of fisetin on allergic contact dermatitis via regulating the balance of Th17/treg in mice. Comput. Math. Methods Med..

[49] Elmehy DA (2021). Amelorative and hepatoprotective effects of fisetin on acute murine toxoplasmosis. J. Egypt. Soc. of Parasitol..

