# Supplementary material for: Evaluation of the anti-inflammatory activity of fisetin-loaded nanoparticles in an in vitro model of osteoarthritis
Source: Sci Rep. 2023 Sep 19;13:15494. doi: 10.1038/s41598-023-42844-1 (PMC10509168; doi:10.1038/s41598-023-42844-1)
Supplement: Supplementary file 1 — Supplementary Information. [file 41598_2023_42844_MOESM1_ESM.docx]

Supplementary Information

**Evaluation of the anti-inflammatory activity of fisetin-loaded nanoparticles in an in vitro model of osteoarthritis**

Zahra Nabizadeh, ^a^ Mahmoud Nasrollahzadeh, ^b^ Ali Akbar Shabani, ^a^ Majid Mirmohammadkhani ^c^ and Davood Nasrabadi ^a, *^

^a^ Department of Medical Biotechnology, Faculty of Medicine, Semnan University of Medical Sciences, Semnan, Iran

^b^ Department of Chemistry, Faculty of Science, University of Qom, Qom 37185-359, Iran

^c^ Department of Epidemiology and Biostatistics, Faculty of Medicine, Semnan University of Medical Sciences, Semnan, Iran

*** Corresponding author.**

E-mail address: [davood.bn@gmail.com](mailto:davood.bn@gmail.com) (Davood Nasrabadi)


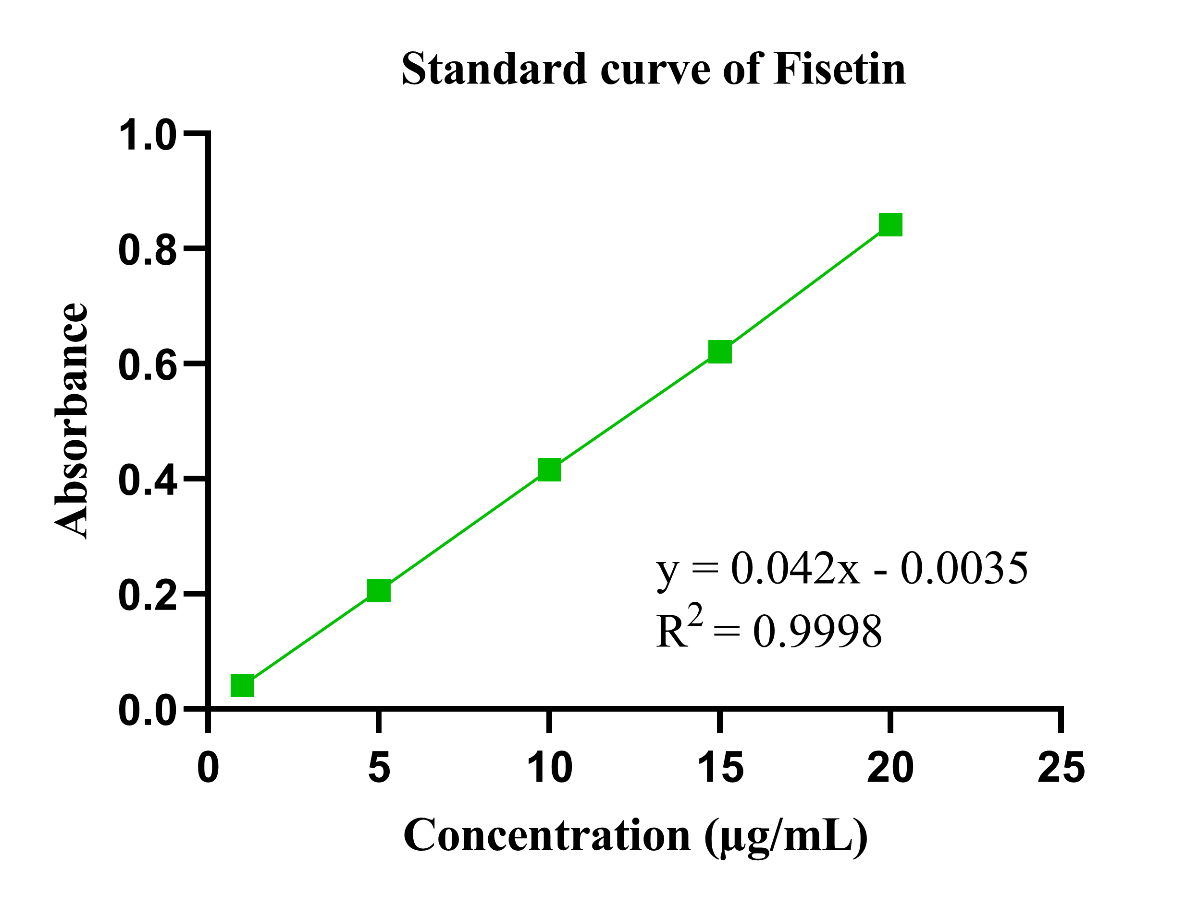


**Figure S1.** Standard curve of fisetin at 364 nm.


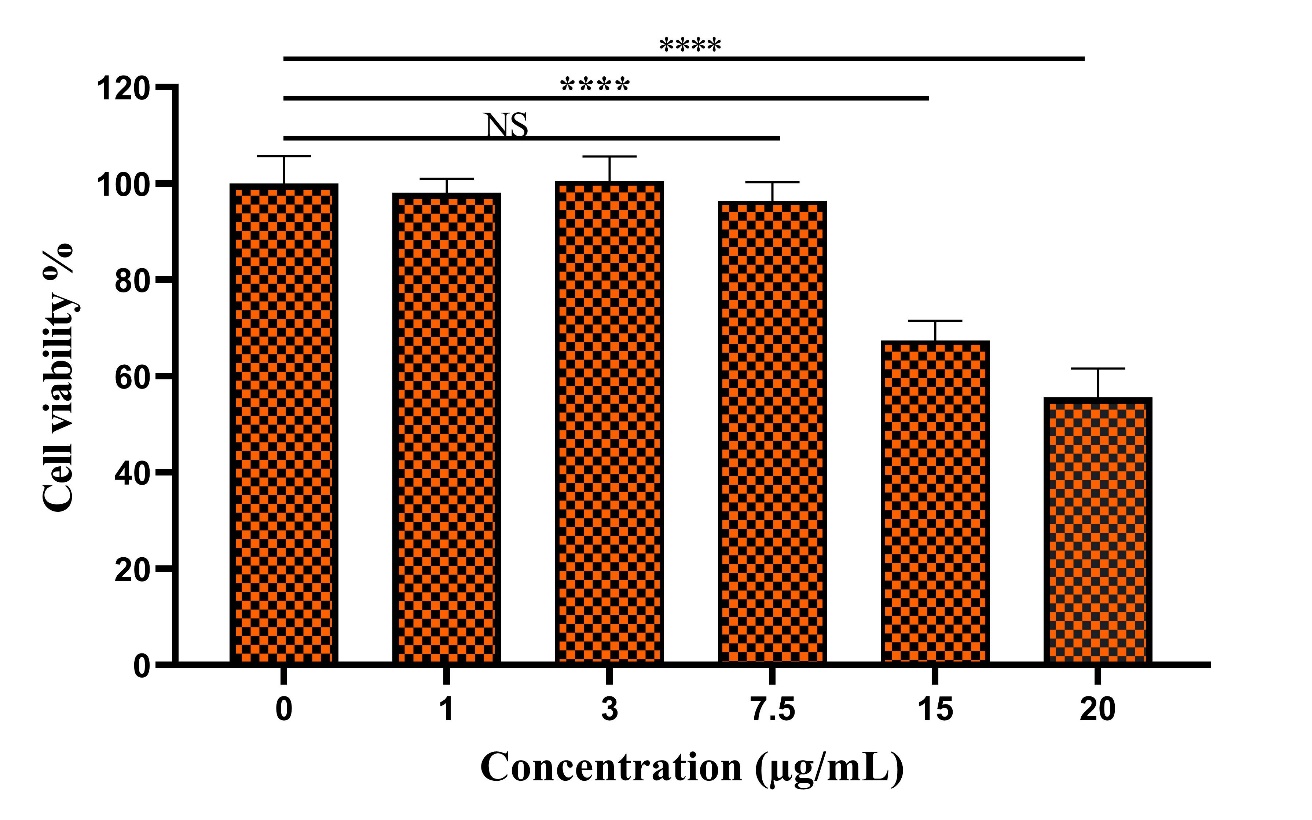


**Figure S2.** The effect of different concentrations of free fisetin on the viability of human chondrocytes. ****p < 0.0001. NS: not significant.
